# Land as a binding constraint to cluster-based development in Ethiopia: To cluster or not to cluster?

**DOI:** 10.1371/journal.pone.0298784

**Published:** 2024-04-16

**Authors:** Guyo Godana Dureti, Martin Paul JR. Tabe-Ojong

**Affiliations:** 1 Ethiopian Agricultural Transformation Agency, Addis Ababa, Ethiopia; 2 Land Economics Group, Rheinische Friedrich-Wilhelms-Universität Bonn, Bonn, Germany; 3 International Food Policy Research Institute (IFPRI), Cairo, Egypt; 4 World Bank, Washington, DC, United States of America; PLOS ONE, UNITED KINGDOM

## Abstract

**Introduction:**

As one of the agglomeration models targeting cluster-based rural development, cluster farming has been promoted in Ethiopia and it is already reported to have significant welfare implications, but participation rates are not as high as expected. This study examines the role of land as a constraint to the development of cluster-based development in Ethiopia both using extensive and intensive measures of cluster farming. The study further disaggregates farm households based on their farm size to better understand potential heterogeneities in the relationship between farm size and cluster farming. The paper also documents other household socio-economic and network characteristics that may matter in cluster farming.

**Methods:**

We use a large-scale farm household data from 3,969 households coupled with some expert insights on cluster farming in Ethiopia. Households in the study areas grow major staples such as maize, wheat, teff, malt barley, and sesame in four main regions of Ethiopia. We employ a double hurdle model to examine both the decision to participate and the extent to which households participate in cluster farming. By extent of participation, we refer to the amount of land and share of land farm households contribute to cluster farming. For robustness purposes, we also estimate the Tobit and Linear Probability Models.

**Results:**

We show a positive association between farm size and cluster farming both at the extensive and intensive margins. This relationship turns negative for large amounts of land. This shows that cluster farming increases with farm size up to a threshold beyond which it declines. We also find suggestive evidence that participation rates are lower for small-scale farms, but also declines for large-scale farms. In addition, we show that access to information and network characteristics also matter in enabling cluster farming.

**Conclusion:**

The findings of this study are relevant in the framework of plans to upscale the cluster-based development initiative in Ethiopia. Attention to landholding issues is key and may be an important area where policy action can be geared to boost cluster farming. Moreover, our results inform potential targeting plans that aim to increase the participation of small-scale farmers who are usually the intended targets of such programs.

## 1. Introduction

In many developing countries, the potential for sustainable growth lies in the agricultural sector, particularly in smallholder farming [1]. Yet, smallholder farmers in developing countries often suffer from constraints such as poor infrastructure, limited access to effective extension and credit services, high per-unit transportation costs, and low bargaining power [2, 3]. Some of the key institutional mechanisms through which smallholders strive to overcome such high transaction costs and market failures is by pooling their resources and efforts together collectively or in groups [4–6]. A group of smallholders can pool their resources together and market their products jointly, thereby reducing transaction costs, lowering information asymmetries, and improving their bargaining power [3, 7]. Organized smallholder farmers can also serve as a useful platform for implementing development projects, disseminating extension information and services, linking to input and output markets, and accessing capacity building and innovations [3–5]. Moreover, grouping of smallholder farmers is an efficient mechanism for extension services and private companies to reach and interact with multiple farmers and share agriculture-related information in a single effort [8, 9].

Cluster farming, which refers to the concentration of agricultural activities creating income and employment opportunities in and around a particular region, represents a new development initiative used by governments and development agencies as a way of increasing collective action among smallholder farmers [1, 10, 11]. As one of the agglomeration models targeting cluster-based rural development, cluster farming is usually geared at increasing farm production and productivity with far-reaching implications on smallholder livelihoods and welfare [8, 12–15]. Cluster farming has also been shown to associated with technology adoption [8, 15] and smallholder commercialization [11] with ensuing income increases and poverty reduction [10, 14]

In the context of Ethiopia, cluster farming approach has been introduced during the Growth and Transformation Plan (GTP I) as Agricultural and Commercialization clusters (ACC), an overarching approach targeting specific geographical locations and different high-value and high-acreage crops throughout the country [16, 17]. Within the ACC, the Farmer Production Clusters (FPCs) was also introduced as a more nuanced intervention aimed at achieving economies of scale by grouping between 30 to 200 smallholder farmers who own adjacent lands [17]. Here, the term ’cluster farming’ refers to these Farmer Production Clusters (FPCs). Under the FPC initiative, smallholder farmers with adjacent farm plots voluntarily pool a portion of their land to benefit from targeted government support and economic agglomeration [10, 17]. Although cluster approach has been promoted and implemented in some regions of the country since 2015/2016, smallholders participation rates in these clusters remains low, with only 7.2% of the country’s 18 million farmers engaged in agricultural production being part of cluster farming as of 2020 [17–19]. Cluster farming also accounts for only 7.21 percent of total land cultivated in Ethiopia [17–19]. Despite the government’s efforts to spread the cluster farming approach throughout the country, farmers may face a variety of constraints that curtail their participation in cluster farming including access to land.

Given this, we examine the relationship between landholding size and cluster farming initiative in Ethiopia. Ethiopia is an ideal setting to understanding this relationship for two key reasons. In the first place, the country is following the cluster-based development approach and rolling out farm clusters in many regions in the country [10, 11, 16, 17]. Second, the cluster farming approach is centered on the land access given that farm households are required to contribute at least 0.25 ha of land to participate in cluster farming [10, 16, 17]. Nevertheless, Ethiopia is facing an escalating issue of land scarcity attributable to the rising population pressure, land fragmentation, and degradation [20–22]. Land degradation is also closely linked to intensified soil erosion, deforestation, and non-sustainable farming practices, compounded by the uncertainty of land ownership rights. Furthermore, Ethiopia’s rural land markets are still underdeveloped, with significant imperfections and market failures [22–24]. In this context, it may be difficult for smallholder farmers to gain access to land to benefit from land ownership-based initiatives such as cluster farming. This may eventually limit farm income and household food security especially given secure and equal access to land is considered key to reducing rural poverty and stimulating rural development in developing nations [25–27].

We use a large-scale farm household data from 3,969 households coupled with some expert insights on cluster farming in Ethiopia to examine the role of land access as a constraint to the development of cluster farming in Ethiopia. We do so in two steps by understanding the role of land access on participation in cluster farming at (1) an extensive margin and (2) intensive margin. We examine the relationship between farm size and cluster farming using a double hurdle model. By extensive margin, we refer to an indicator variable capturing cluster farming while the extent of participation which refers to the amount of land allocated to the clusters is the intensive measure of participation. We hypothesize a non-monotonic relationship between farm size and cluster farming. Beyond this, we disaggregate households based on their landholding and examine the association between farm size and the extensive and intensive measures of participation. We also document the importance of other farm level, household level and cluster level characteristics.

We show that farm size is positively associated with cluster farming, both at the extensive and intensive margins. However, the results become negative for large farm sizes. This suggests that cluster farming is increasing with farm size up to a threshold point (~5.67 hectare) beyond which it declines. This relationship suggests that land may be a binding constraint to the participation of farmers in farm clusters. We also show that other characteristics matter in cluster farming, particularly human capital related characteristics, household access to information and social networks, and cluster-level characteristics. Our findings are robust to other nonlinear models like the Tobit model and linear models like ordinary least squares in the framework of the linear probability model.

Previous studies on cluster farming have primarily focused on the role of cluster farming in improving marketing conditions and welfare, with little attention paid to various constraints faced by farm households to participate in cluster initiatives [11–14, 28]. Zhang and Hu [12] show that potato agro-clusters improve potato production and foster rural development in China. Montiflor et al. [13] highlight that vegetable agro-clusters improve access to farm inputs and increase market surplus in the Philippines. Agro-clusters have also been shown to reduce poverty in Indonesia [14, 28], and in Ethiopia [10]. Goetz et al. [29] also highlight the profitability and productivity implications of clusters in the United States. In India, the “Hub-and-spoke” cluster model has been shown to create greater agglomeration economies in ways that support smallholder farmers and offer stable inputs to Agro-Industrial Parks [30]. Aside from crop benefits, agro-clusters have been demonstrated to boost fish production, allowing farmers to reap the benefits of economies of scale in Fiji [31] and improve networks and promote adoption of aquaculture practices in Vietnmen [15].

We add to these studies on cluster farming in several ways. In first place, we look at the role of land access as a constraining factor in the cluster participation process. Here we aim to provide policy implications for how the benefits of cluster farming observed in various studies can be sustained through improved cluster farming practices. Second, we show that land access could be a critical entry point for policy to boost cluster farming in Ethiopia. Although participation in cluster farming is dependent on landholding, access to landholding is decreasing due to population pressures as well as imperfect rural land markets, suggesting that policy interventions may be required to increase participation in cluster farming. Third, we argue that although land matters for the development of cluster farming, very large farm sizes may reduce participation in these clusters. This finding implies a potential diminishing return when farm sizes exceed 5.67 hectares, which could mean that farmers with larger land parcels may find cluster farming less beneficial or manageable. This also suggest that the growth and sustainability of cluster farming may need to consider a balance between size and efficiency in farming operations.

Fourth, we look at cluster farming in various staple crops such as wheat, teff, malt barley and sesame which are all crops grown by smallholder farmers with implications for food security and poverty. The cultivation of these crops can be described as inclusive since they are usually cultivated by resource poor smallholder farmers [10]. Finally, our analysis contributes to the understanding of the land debate in Ethiopia and its implications for various developmental initiatives with implications for smallholder livelihoods and welfare.

The rest of the study is organized as follows. Section 2 briefly discusses the concept of clustering and its overview in Ethiopia. Section 3 outlines the theoretical framework and econometric procedures used to estimate smallholders’ participation decision and extent in cluster farming. In addition, it discusses the data used in the study and the summary statistics of the variables used in the empirical analysis. The results and discussion section 5 provides and discusses the estimated determinants of smallholder participation decision and intensity. The last section 6 summarizes the main findings and draws some policy implications and outlook for further research.

## 2. Background and context

### 2.1 Land access in Ethiopia

To understand the role of land in cluster farming, we begin by highlighting the context under which this initiative operates. We look at the mechanisms of gaining access to land, such as government redistribution policies, norms of inheritance, and the different patterns of land market in the country.

Ethiopia has witnessed numerous forms of land tenure system under different government regimes, which differently affects access to land in the country. During the imperial era (1930–1970), land was predominantly concentrated in the hands of absentee landlords, posing significant threats to tenant farmers who faced arbitrary evictions [21, 23]. The land tenure system went under fundamental reforms during the Derg socialist regime (1970–1991), where land ownership shifted to the state, transforming the agrarian structure with increasing access to land [21, 32]. However, after the fall of the Derg, land issues were largely neglected in the reform process, and the legacy of the Derg regime continued to dominate the present land policy environment [21, 33, 34].

Currently, land in Ethiopia is considered public property, and farmers only have usufruct rights, with no rights to sell, mortgage, or exchange land [22, 35]. Aside from inheritance, other possible ways of acquiring access to rural land in Ethiopia are through fixed-rental and sharecropping arrangements [20, 22, 35]. In fixed land rental contracts, the tenant pays a fixed rate and assumes the right to use the land and harvest during the agreed-upon production season. Harvests are shared between tenants and landlords in sharecropping arrangements, where tenants agree to share a portion of harvested production with the landowner. But these are still primarily limited to urban and peri-urban areas.

On the other hand, some changes were made by the current government (post derg regime), including devolving responsibility for land issues to regions, delaying the frequency of land redistribution, and allowing an informal land market with some restrictions [34]. Despite some of these changes, Ethiopia still grapples with severe land scarcity particularly with increasing population pressure leading farm sizes to dwindle [21, 34]. Furthermore, other land degradation issues such as soil erosion, nutrient depletion, and deforestation aggravate the situation. In countries with limited expanses of land and governed by usufruct land rights systems, such as Ethiopia, the primary avenue for people (mostly the young) to acquire land is through periodic land redistribution, inheritance, and informal rental markets [21, 34]. The implications of access to land are thus crucial to document, particularly for innovative policy programs such as cluster farming whose functioning hinge on access to land.

### 2.2 Cluster farming in Ethiopia

In the agricultural sector, the concept of a ’cluster’ generally refers to the aggregation of agricultural activities that spur income generation and employment opportunities within and around a designated regions [36]. This concept is further defined as a collection of producers, agribusinesses, and institutions that operate within the same agricultural or agro-industrial segment, consolidating value networks while addressing collective challenges and harnessing collective opportunities [1]. It is also increasingly used as a developmental tool to achieve agricultural transformation in many developing countries [1, 10].

In Ethiopia, the ’cluster’ concept has been introduced during the first Growth and Transformation Plan (GTP I, 2010–2015) as the Agricultural Commercialization Clusters (ACC), a government-implemented policy intervention targeting specific geographical regions and selecting both high-value, and high-acreage crops across the nation [16, 17]. ACC endeavours to overcome the critical challenges of scale and inadequate smallholder integration through production and productivity augmentation, while also fostering and integrating commercialization initiatives [16, 17]. Specifically, the ACC enhances commercial prospects for smallholders by intensifying the quantity and quality of agricultural inputs (such as fertilizer, seed, credit, mechanization, etc.) and linking them with output markets [16, 17].

Under the ACC, the Farmer Production Cluster (FPC) was introduced as a more nuanced intervention aimed at achieving economies of scale by grouping between 30 to 200 smallholder farmers who own adjacent lands [17]. The goal of this intervention is to assist smallholder farmers in transitioning from purely subsistence farming to a model that incorporates commercial activities, achieved through two mechanisms [17]. First, it enables smallholders possessing adjacent land to cooperate, thus attaining economies of scale which leads to affordability of modern technology (e.g., sharing tractor purchasing costs), robust bargaining power (e.g., negotiating favorable product prices), enhanced market linkages to cater to bulk or large-scale buyers (e.g., contract farming with major processors), and swift adoption of best farming practices and extension services. Second, it enables systematic and coordinated implementation of a variety of government interventions along crop value chains including provision of elementary inputs (e.g., fertilizers, improved seeds, credit, and mechanization), storage and transport facilities, and market linkages (e.g., contract farming). Participation in these clusters by smallholder farmers is voluntary and subject to the fulfilment of three primary conditions [17]: (1) they must contribute at least 0.25 hectares of land; (2) the total land contributed by a cluster must be at least 15 hectares and (3) participating farmers must be willing to cultivate priority crops as determined by the cluster and adhere to the cluster’s recommendations.

### 2.3 Conceptual framework of land access and cluster farming

Given participation in cluster farming requires access to land, in our conceptual framework, we posit that farm households make two sequential decisions regarding participation in cluster farming. The first decision revolves around whether to participate in cluster farming. As participation is voluntary, it is expected that households that exhibit willingness to integrate into cluster farming will decide to join and allocate a certain plot size for these purposes. The potential constraining factors are a lack of awareness and understanding of cluster benefits, as well as a lack of access to land to contribute to cluster membership. In the second stage, the household determines the actual quantity of land or the proportion of land to be contributed to cluster farming. The amount of land contributed could potentially influence the extent of participation and the potential benefits gained from cluster farming activities, thus making these paired decisions central to understanding the dynamics of cluster farming adoption and implementation.

For the empirical specification, we follow the literature on participation in producer organisations in rural settings as this is similar to our case [e.g. 2, 28, 37, 38]. These literatures highlight that farm households could make two subsequent decisions to take part in group activities; whether to participate and to what extent to participate if they do participate. Here, we assume that different households perceive the costs and benefits of participation in cluster farming differently, and the main decision is made at the household level. We formally represent this in a random utility framework, where a representative household is expected to participate in cluster farming to maximize its underlying utility. This means that the households participate in cluster farming if the expected net benefit from participation is greater than non-participation, as shown in the following model:

$$C_{i}^{*}=\theta Z_{i}+\mu_{i},\;\mathrm{with}\;D_{i}=\left\{ \begin{array}{c} 1\;if\;C_{i}^{*}>0,\\ 0\;otherwise \end{array}\right.$$ (1)

$C_{i}^{*}$ is a latent variable indicating the utility difference between participating and not participating in cluster farming; *C*_*i*_ is an observable binary variable that takes value 1 if household participates in cluster farming and 0 otherwise; **Z**_**i**_ is a vector of exogenous variables, ***θ*** is a vector of parameter estimates, and *μ*_*i*_ is stochastic error term which represents the unobservable part of a smallholder utility function. Based on this, the probability that a smallholder farmer participates in cluster farming is derived and estimated as follow:

$$P_{r}(C_{i}=1)=P_{r}(C_{i}^{*}>0)=P_{r}(\mu_{i}>-\theta Z_{i})=1-F(-\theta Z_{i})$$ (2)

## 3. Methods and materials

The farm household’s two-steps participation decisions can be modelled either jointly or separately [39]. In most cases, either the restrictive one-tier Tobit model [e.g. 40, 41], or the relaxed two-tier model variants such as Heckman sample selection [e.g. 42], or Double Hurdle model [e.g. 14, 43] are employed.

The Tobit model is a left-censored approach that models household decisions as a single decision-making process by assuming simultaneous decisions on participation and intensity [44]. It assumes that the household decision process is determined by the same equation and variables [44]. The drawback of using this model is based on its fairly restrictive underlying assumption, especially when there is two step decision-making procedure, with different factors having separate and unique correlation with the decision to an extent to participate [39]. Moreover, it is less suitable where different independent variables are expected (used) to model the two participation stages.

The Double Hurdle (DH) is a two-tier model that relaxes the restriction in the Tobit model by allowing different mechanisms to determine the discrete probability of participation and intensity of participation [39]. It allows sequential decisions (i.e., the decision to take up initiative preceding the decision on extent), and the same factors can influence the decision and level of participation in different ways. Similarly, different factors can be used to model each stage. This is the case in this study since, for example, cluster size and the number of farmers per cluster may be related to participation intensity but not the initial decision to participate or not. This is because cluster characteristics are only attributable to farm households actively participating in cluster farming. However, for cluster participants, the size of the cluster and the number of farmers may be among factors that may influence the amount of land a particular household contributes to cluster farming. The DH model uses the corner solution to treat zero values associated with non-participation as the outcome of household rational choice, rather than by incidence [45]. Hence, we use the DH model developed by Cragg [46] for this analysis.

### 3.1. Double hurdle model

The Cragg model uses a probit model in the first stage to determine the probability of participation and a truncated model in the second stage to evaluate the factors that influence the level of participation conditional on the participation decision. A general form of Cragg’s Double hurdle model can be formulated as below.

Following Eq (1) above, let *D*_*i*_ be an observed binary variable indicating household *i*^th^ participation decision in cluster farming while $D_{i}^{*}$ represents a corresponding latent variable. The first hurdle (probit) model can be specified as:

$$D_{i}^{*}=X_{i}\alpha +u_{i}$$ (3)

$$D_{i}=\left\{ \begin{array}{c} 1\;if\;D_{i}^{*}>0,\\ 0\;otherwise \end{array}\right.$$

Where, ***X***_***i***_ is a vector of explanatory variables; ***α*** is a vector of parameters to be estimated and *u*_*i*_ is the error term.

Again, let *y*_*i*_ be a non-negative continuous variable that represents the amount of land (or share) of land *i*^th^ household contributes to cluster farming and $y_{i}^{*}$ is a corresponding latent variable. The second hurdle (truncated model) can be specified as:

$$y_{i}^{*}={FS}_{i}\delta +{FS}_{i}^{2}\vartheta +X_{i}\beta +v_{i}$$ (4)

Where *FS*_*i*_ and *FS*_*i*_^2^ refer to farm size and the square of farm size respectively. Our parameters of interest are *δ* and *ϑ* which represents coefficients of farm size and the square of farm size respectively. Farm size refers to the number of hectares of farmland “owned” by a household. Farm size could be an important limiting factor of production, particularly in the context of this study, given households are required to contribute at least a quarter hectare of their land to participate in cluster farming. As such, we expect that farmers with relatively larger land size may be more likely to participate in cluster farming and contribute more land. On other hand, land is considered to be exogenous and de facto owned by the household [35]. This could imply that, while households with large land endowments may allocate more land to cluster farming and benefit more from the associated gains of pooling resources and economies of scale advantages, these more advantaged households may not obtain more land to allocate in return due to exogenous land ownership. However, households that allocate most or all their land to cluster farming may have little or no additional land to allocate. Thus, their participation in cluster farming may be eventually limited by size of their farm. ***X***_***i***_ is a vector of control variables influencing the cluster farming and ***β*** are their parameter estimates, and *v*_*i*_ is the error term. The choice of control variables is guided by the theoretical framework and insights from the empirical literature [e.g. 2, 37, 47–51]. Potential variables include household socioeconomic characteristics, institutional and social factors, cluster characteristics, and environmental and ecological conditions. We also use different controls in the participation decision model (Eq 3) and extent of participation model (Eq 4). While the second stage truncated model includes all explanatory variables, the first stage participation decision model excludes the two variables related to cluster farming characteristics ((i.e., cluster land size and number of cluster members). This is because these variables only observed for our second equation sample, only participating households, given that non-participants are not associated with any cluster farming.

Following Carroll et al. [52] and Mal et al. [53], Eqs (3) and (4) are assumed to be uncorrelated and the two error terms are randomly and independently distributed as:

$$\left(\begin{array}{c} u_{i}\\ V_{i} \end{array}\right)∼N\left[(\begin{array}{c} 0\\ 0 \end{array}),(\begin{array}{c} 1\;0\\ 0\;\sigma^{2} \end{array})\right]$$ (5)

The Cragg double-hurdle model is estimated using the maximum likelihood estimation (MLE) technique. First, MLE in hurdle I is obtained from Probit estimator, then the MLE for hurdle 2 is estimated from the truncated normal regression model. The consequent log-likelihood function model can be specified below following Cragg [46] and Mal et al. [53]:

$$LL=\sum_{0}ln[1-\Phi (w_{i}^{\prime }\alpha )\Phi (\frac{x_{i}^{\prime }\beta }{\sigma })]+\sum_{+}ln[\Phi (w_{i}^{\prime }\alpha )\frac{1}{\sigma }\emptyset (\frac{y_{i}-x_{i}^{\prime }\beta }{\sigma })]$$ (6)

Where, Φ is the standard normal cumulative distribution function and ∅ is the density function.

The double-hurdle model is reduced to the Tobit model if each household is assumed to participate in FPC (i.e., $\Phi (w_{i}^{\prime }\alpha )=1$), which implies both participation decision and the intensity of participation are made simultaneously. For appropriate modelling and robustness, the likelihood ratio (LR) test of DH against the Tobit model is performed as:

$$LR\;static=-2[lnL_{T}-(lnL_{P}+lnL_{TR}]$$ (7)

Where L_*T*_ is the likelihood of Tobit, *ln*L_*p*_ is the likelihood of probit and L_*TR*_ is the likelihood of truncated regression. *LR static* has a *χ*^2^ distribution.

## 4. Data and descriptive statistics

### 4.1 Household survey

The study makes use of a large-scale farm household survey conducted in February 2020 and April 2021 in Ethiopia. The survey included farm households producing Ethiopia’s main staple crops like wheat, maize, teff, barley, and sesame in four major regions (Amhara, Oromia, Southern Nations, and Tigray). The two rounds of surveys were conducted by Ethiopian Agricultural Transformation Agency as part of the assessment of the performance of cluster farming during both periods. The survey was designed and administered on survey-based tablets, which enabled real-time quality checks and controls. The interviews were also carried out by a group of well-trained enumerators. Participants were informed about the survey for consent and verbal consents from each participant were obtained before proceeding with the interviews. The surveys captured information on the household socioeconomic characteristics and value chain activities. Specifically, the survey included household socio-demographic characteristics (gender, age, education, and family size), household farm assets (land size, off-activities, total production, and market surplus output), and social network (neighbour participation in cluster farming, awareness, and membership in self-help groups). Information was also captured on access to extension services and credit.

Regarding sampling method, a multistage sampling technique was used to select households in the two survey periods. In the first stage, 75 total woredas (including 50 cluster and 25 non-cluster woredas) were randomly selected proportional to size. Woredas where cluster farming has been promoted are our treatment woredas whereas woredas where cluster farming does not exist at the time of the survey form our control woredas. The treatment and control woredas are similar in terms of farming systems and practices, cultivate similar crops, and belong to similar agro-ecological zones, except that the cluster farming has not been promoted in control woredas. Our data also shows that we do not record any participation in cluster farming from the control woredas. However, some of the control woredas are areas where the government intends to scale up the cluster approach, but at the time of the survey, no clusters have been established. Given that households were randomly selected in the treatment woredas, about 25% of households interviewed in treated woredas did not participate in agro-clusters. From these 75 woredas, kebeles were randomly selected and households were further randomly selected for interviews. In total, we reached 3978 households over the two survey periods, but due to some missing entries, we only used 3969 household data.

Although we have a two-period data, these data cannot be treated as a panel as different households were interviewed in each year, so we treat our data as cross-sectional. However, we include year dummies in all regressions to control for year effects. We also add regional dummies and cluster around woreda to account for agro-ecological and farming system differences in the study sites

### 4.2 Variables description and descriptive statistics

Table 1 presents the summary statistics for all variables used in this study. We have three outcome variables: (1) participation in cluster farming, (2) amount of land households contributes to cluster farming, and (3) share of land household contributed to cluster farming. The first outcome variable is the dependent variable in the first stage of the double-hurdle model, and it is a dummy variable that takes a value of 1 if the household head participates in cluster farming and 0 otherwise. The second and third variables are dependent variables in the second-stage hurdle model and are proxies for the extent of participation. We use amount of land contributed as main variable of interest for the second hurdle and perform robustness checks with the share of land contributed. The household participation dummy indicates about 57% of households in the study area participate in cluster farming. These households allocate an average of 0.60 hectares of land to the cluster farming and their contributed land is about 27% of their total landholding on average. There are about 17 household members per cluster, and the total land per cluster is about 12 hectares.

**Table 1:** Summary statistics.

	Full sample	Cluster (participant)	Cluster (non-participant)	Control (non-participant)
***Dependent variables***				
Cluster farming (dummy)	0.57 (0.50)	1.00 (0.00)	-	-
Plot allocated to cluster (ha)	0.60 (0.87)	1.06 (0.92)	-	-
Plot allocation ratio (0–1)	0.27 (0.31)	0.490 (0.263)	-	-
***Control variables***				
Age of household head (years)	42.67 (11.02)	41.99 (10.22)	42.53 (11.95)	45.05 (11.80)
Primary education (dummy)	0.73 (0.44)	0.814 (0.389)	0.651 (0.477)	0.588 (0.492)
Household head is female (dummy)	0.09 (0.29)	0.108 (0.311)	0.0861 (0.281)	0.0890 (0.285)
Household size (number)	6.51 (2.44)	6.644 (2.430)	6.302 (2.454)	6.380 (2.465)
Landholding (hectares)	2.25 (1.96)	2.540 (2.227)	1.890 (1.465)	1.844 (1.470)
Group membership (dummy)	0.37 (1.96)	0.388 (0.487)	0.331 (0.471)	0.405 (0.491)
Credit access (dummy)	0.33 (0.47)	0.515 (0.500)	0.0892 (0.285)	0.167 (0.373)
Extension access (dummy)	0.90 (0.29)	0.973 (0.162)	0.816 (0.388)	0.814 (0.390)
Storage facilities (dummy)	0.59 (0.49)	0.673 (0.469)	0.617 (0.486)	0.334 (0.472)
Off-farm income (dummy)	0.40 (0.49)	0.434 (0.496)	0.318 (0.466)	0.451 (0.498)
Total cluster size (hectares)	11.46 (16.88)	20.10 (18.06)	-	-
Cluster members size (number)	16.63 (24.56)	29.17 (26.31)	-	-
Wheat (dummy)	0.303 (0.460)	0.303 (0.460)	0.293 (0.455)	0.284 (0.451)
Teff (dummy)	0.143 (0.350)	0.143 (0.350)	0.139 (0.346)	0.138 (0.345)
Sesame (dummy)	0.0614 (0.240)	0.0614 (0.240)	0.0760 (0.265)	0.113 (0.316)
Barley (dummy)	0.147 (0.354)	0.147 (0.354)	0.131 (0.337)	0.145 (0.352)
Maize (dummy)	0.346 (0.476)	0.346 (0.476)	0.362 (0.481)	0.321 (0.467)
Awareness of Cluster farming (dummy)	0.22 (0.42)	0.961 (0.193)	0.615 (0.487)	0.441 (0.497)
Neighborhood participation (dummy)	0.78 (0.41)	0.244 (0.430)	0.285 (0.452)	0.106 (0.308)
Observations	3,969	2,263	987	719

Notes: Standard errors are in parentheses

Among the household socio-economic characteristics, the average age of the household head is 43 years. Most households are male headed with average family size of 6 members. Education, on average, 73% of households have access to primary education level. Average total landholdings are about 2.25 hectares. Looking at household at different farm size, while about 30% of households own land size less than 1 hectare, about 60% of household farm size ranges between 1 and 5 hectares. This shows that most households are smallholder farmers although the average farm size is larger than the national average of 1 hectare per household. Regarding institutional factors, extension access in the study area is widespread (90%), and 33% of household heads have access to credit facilities.

Looking at access to information and social networks, awareness about cluster farming is relatively high with about 85.65% of households reporting to have a clear understanding of cluster farming and how it operates. According to the survey, households learn about cluster farming from three primary sources: Government officials promotion activities, Development Agents (DAs) campaigns, and interactions with neighbouring participating households. Other social network variable is neighbour participation which refers to whether a household is aware of the participation of their neighbour. On average, 22% of households are aware of their neighbour’s participation in cluster farming. Cluster-level characteristics show cluster farming is about 11.45 ha and about 17 farm households form clusters.

We also further disaggregate farm households into three groups (the last three columns of Table 1): cluster participants, non-participants from cluster woredas and non-participants from control woredas. Looking at average farm size of households from the three groups, cluster participants have 2.54 hectares, but non-participants from cluster villages and control villages have 1.89 and 1.84 respectively. The awareness of cluster farming (knowing its existence and understanding of the concept) also differs between these groups ranging from 96% for cluster participants to about 62% of non-participants from cluster villages and 44% of non-participants from control villages.

To understand observable differences between cluster farming participating and non-participating households, a mean difference test is conducted and presented in Table 2 below. The result shows that there are significant observable differences between the two groups. The household demographic characteristics shows that participant group is led by the younger and relatively more educated head of household, with the difference being significant at 1%. The two groups also differ significantly in terms of land owned, off-farm income, value of crop produced, market surplus and commercialization index, with each result significant at a 1% level. On average, landholding is 2.54 hectares for participants and 1.88 hectares for non-participants.

**Table 2:** Mean differences based on participation in cluster farming.

	Participants	Non-participants	t-test
Land holding (hectares)	2.54 (0.04)	1.87 (0.03)	***
Age of household head (years)	41.98 (0.21)	43.59 (0.28)	***
Primary education (dummy)	0.81 (0.01)	0.62 (0.01)	***
Household size (number)	6.64 (0.05)	6.33 (0.05)	***
Extension access (dummy)	0.97 (0.00)	0.82 (0.01)	***
Credit access (dummy)	0.51 (0.01)	0.12 (0.00)	***
Awareness of Cluster farming (dummy)	0.96 (0.00)	0.54 (0.01)	***
Neighborhood participation (dummy)	0.24 (0.01)	0.21 (0.01)	***
Observations	2,263	1,706	

Notes: Standard errors are in parentheses

*** p<0.01

** p<0.05

* p<0.1.

## 5. Results and discussion

### 5.1. Estimates of farm size and cluster farming

As mentioned earlier, the double-hurdle model is an alternative to the Tobit specification, since the latter is nested in the former. To compare the two models for a suitable specification, we perform a likelihood ratio as shown in Eq (7). The statistical result of LR is 331.99, with a chi-square probability of (*Prob*>*χ*^2^ = 0.00). Hence, the null hypothesis that smallholder farmers’ participation in cluster farming with one decision can be rejected in favour of the two-step decision process of the double hurdle model. After estimating the double hurdle model, the average marginal effects (AME) are obtained, which are reported in regression results.

Table 3 presents the results of the maximum likelihood estimation of the double hurdle model for the probability of participation in cluster, and amount and share of land contributed conditional on participation. The result of Tobit model is also reported in the same table, showing the similar finding which confirms the robustness of our findings. For simplicity, we discuss all results in terms of the amount of land contributed specification only. The probit model result in the Table 3 (first column) shows that farm size is positively and significantly related to the probability of participating in cluster farming. On average, an additional hectare of landholding increases the likelihood of cluster participation by approximately 5%, all other factors remaining constant. This is plausible given that larger farm households are less constrained by the minimum amount of land required to participate. However, the likelihood of participation decreases with farm sizes greater than 12.5 hectares as implied by the negative coefficient of land size squared. This result is consistent with previous related studies which also show farm size is positively associated with collective action [2, 28], but negatively associated with the square of farm size [2].

**Table 3:** Double Hurdle estimates of land holding and cluster farming.

	HD I	HD II	Tobit Model
	Cluster participation decision Probit estimator	Amount of land contributed to cluster Truncated estimator	Share of land contributed to cluster Truncated estimator	Amount of land contributed to cluster
	AME	AME	AME	AME
Total Landholding size (Ha)	0.047*** (0.008)	0.314*** (0.129)	-0.096*** (0.006)	0.348*** (0.020)
Land holding square (Ha sq)	-0.001* (0.001)	-0.012*** (0.020)	0.004*** (0.000)	-0.009*** (0.001)
Other controls	Yes	Yes	Yes	Yes
Crop dummies	Yes	Yes	Yes	Yes
Region dummies	Yes	Yes	Yes	Yes
Time controls	Yes	Yes	Yes	Yes
Constant	Yes	Yes	Yes	Yes
Observations	3,559	1,853	1,853	3,559

Standard errors in parentheses

*** p<0.01

** p<0.05

* p<0.1

Table 3 (the second column) shows the result of truncated normal regression with respect to the amount of land contributed to cluster farming conditional on participation in the clusters. Here our finding shows landholding is positively associated with the amount of land contributed to cluster farming conditional on participation at a 1% level of significance. Given positive participation, each additional hectare of land increases the amount of land contributed to cluster by about 0.314 hectares, other factors remaining the same. However, the amount of land contributed conditional on participation decreases for farm size larger than 5.76 hectares as indicated by the negative coefficient of landholding squared term. The result implies that conditional on participation, the amount of land household contributes to cluster farming increases at a decreasing rate and decreases beyond threshold. Fischer and Qaim [3] also obtained similar results for smallholders who make their land available for banana cultivation as part of a collective action initiative, but with threshold of 11 acres.

### 5.2. Estimates of heterogeneities of farm size and cluster farming

**Fig 1:**
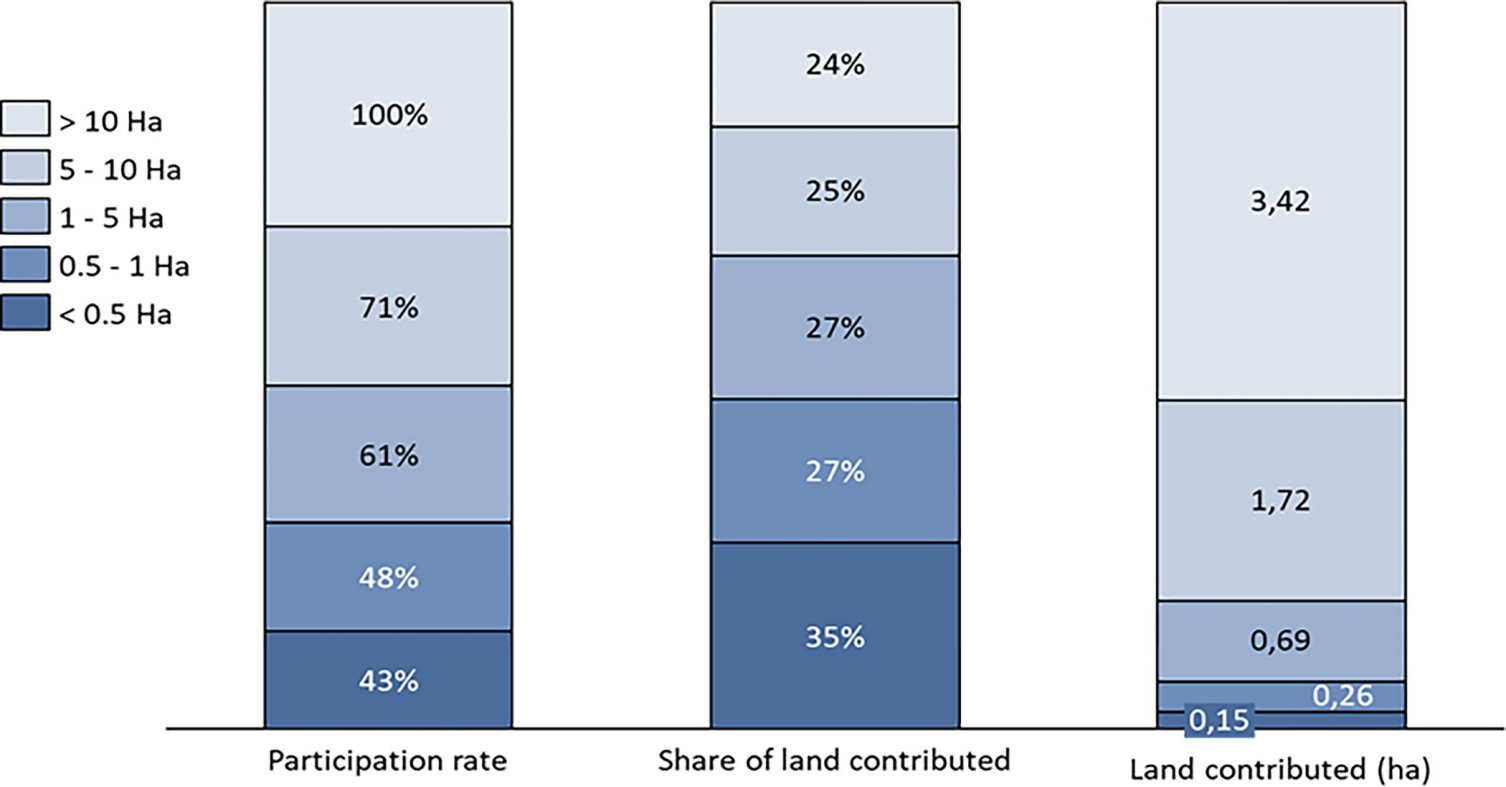
Distribution of farm households based on farm size. The figure illustrates farm households extensive (bar 1) and intensive (bar 1 and 2) participation in clusters in terms of land ownership.

We also categorize farm households into five groups based on farm size. As shown in Fig 1, farm household participation in cluster farming increases from 43.3% for households with less than 0.5 hectares to 70.5% and 100% for farm households with farm sizes of 5–10 hectares and greater than 10 hectares, respectively. Similarly, the average size of landholding contributed by these households increases from 0.15 ha for holders of less than 0.5 hectares to around 3.5 ha for farm households holding more than 10 hectares. However, these households’ share of land contribution does not increase with farm size. Households with less than 0.5 hectare contribute approximately 35% of their holding, while households with more than 10 hectares contribute approximately 23.6%. This result indicates the importance of landholding size in participation of cluster farming.

Furthermore, controlling for other factors, we estimate the effect of landholding on participation and extent of participation using the double hurdle and Tobit models (see Table 4). Our findings show that the effect of land size is insignificant for farm households with less than one hectare, both in terms of probability and intensity of participation. However, when farm size exceeds one hectare, the effect becomes positive and statistically significant. However, the effect of farm size on the probability of participation becomes negative once again when farm size exceeds 10 hectares. Therefore, our results reveal that access to landholding maybe one of key constraint for low participation of farm households in cluster farming.

**Table 4:** Estimates heterogeneities of land holding and cluster farming.

	HD Model		Tobit Model
	(I)	(II)	(III)
**Small farm size (≤0.5ha)**			
Total Landholding size (Ha)	-1.890	-0.267	-1.013
	(4.071)	(1.487)	(2.507)
Land holding square (Ha sq)	2.076	1.140	1.704
	(5.492)	(1.993)	(3.376)
Observations	253.000	144.000	385.000
**Small medium farm size (0.5–1 ha)**			
Total Landholding size (Ha)	6.082	-1.223	3.348
	(5.087)	(8.332)	(7.770)
Land holding square (Ha sq)	-3.475	0.983	-1.709
	(2.921)	(4.778)	(4.459)
Observations	759.000	314.000	759.000
**Medium farm size (1–5 ha)**			
Total Landholding size (Ha)	0.151***	0.814***	0.490***
	(0.045)	(0.129)	(0.115)
Land holding square (Ha sq)	-0.018**	-0.071***	-0.032*
	(0.008)	(0.020)	(0.019)
Observations	2,236.000	1,270.000	2,236.000
**Large farm size (> 5 ha)**			
Total Landholding size (Ha)	-0.349*	0.814***	0.397*
	(0.211)	(0.129)	(0.212)
Land holding square (Ha sq)	0.023*	-0.071***	-0.012
	(0.014)	(0.020)	(0.009)
Observations	179.000	1270.000	179.000
Additional controls	Yes	Yes	Yes
Crop dummies	Yes	Yes	Yes
Region dummies	Yes	Yes	Yes
Time controls	Yes	Yes	Yes
Constant	Yes	Yes	Yes
Observations	3,427	2,998	3,559

Standard errors in parentheses

*** p<0.01

** p<0.05

* p<0.1

### 5.3. Other factors associated with cluster farming

In this section, we discuss other factors associated with smallholders’ participation in cluster farming and potentially be constraints. We particularly focus on household human capital related factors, information and social networks, and cluster farming characteristics.

As shown in Table 5, the probit estimates show that the likelihood of household participation in cluster farming is significantly related to both age and education. The age of the household head is negatively associated with cluster participation. This result may suggest that younger households are more likely to be open and willing to take risks in order to try new initiatives like cluster farming. However, this contradicts the findings of other related studies on cooperative and collective action [e.g., 2, 14, 38, 44]. According to these studies, younger people are more eager for change and new opportunities, whereas older people have more farming experience and social networks, which may increase their likelihood of participating in group initiatives. Fischer and Qaim [3] further adds that the younger generation is less interested in agriculture. On other hand, education variable is positively related to participation in cluster farming, which may suggest that educated household heads may be better able to process and apply new information. Related research indicates that the effect of household education is positive, but insignificant [e.g. 7, 54].

**Table 5:** Estimates of other constraints and cluster farming.

	HD I	HD II		Tobit Model
	Cluster participation decision Probit estimator	Land contributed to cluster Truncated estimator	Share of land contributed Truncated estimator	Land contributed to cluster
	AME	AME	AME	AME
HH head’s Age (Years)	-0.003***	-0.003	-0.002***	-0.007***
	(0.001)	(0.001)	(0.000)	(0.001)
	-0.003***	-0.003	-0.002***	-0.007***
HH primary education (Yes = 1)	0.084***	0.035	0.008	0.183***
	(0.015)	(0.043)	(0.015)	(0.047)
HH is female (Yes = 1)	0.147***	-0.046	0.015	0.262***
	(0.022)	(0.055)	(0.018)	(0.063)
HH family size (Number)	-0.005	0.001	0.002	-0.038
	(0.014)	(0.006)	(0.003)	(0.042)
Neighbour participation (Yes = 1)	0.406***	0.003**	-0.002**	1.176***
	(0.017)	(0.034)	(0.012)	(0.071)
Cluster awareness (Yes = 1)	0.147***	0.226**	0.112**	0.262***
	(0.022)	(0.087)	(0.034)	(0.063)
Group membership (Yes = 1)	-0.002	-0.0042	-0.007	-0.004
	(0.013)	(0.037)	(0.014)	(0.039)
Access to storage (Yes = 1)	0.011***	-0.085**	-0.056***	0.014*
	(0.003)	(0.038)	(0.014)	(0.008)
Access to credit services (Yes = 1)	0.074***	-0.024	-0.0224	0.098**
	(0.014)	(0.032)	(0.012)	(0.042)
Access to extension (Yes = 1)	0.300***	-0.030	-0.029	0.436***
	(0.012)	(0.104)	(0.039)	(0.040)
Off-farm activities (Yes = 1)	0.210***	-0.118	-0.037	0.615***
	(0.028)	(0.032)	(0.012)	(0.097)
Cluster total area size (ha)		0.011***	0.004***	0.028***
		(0.001)	(0.000)	(0.002)
Cluster member size (number)		-0.005***	-0.002***	0.003***
		(0.001)	(0.000)	(0.002)
Crop dummies		Yes	Yes	Yes
Region dummies		Yes	Yes	Yes
Time controls	Yes	Yes	Yes	Yes
Constant	Yes	Yes	Yes	Yes
	Yes			
Observations	3,559	1,853	1,853	3,559

Standard errors in parentheses

*** p<0.01

** p<0.05

* p<0.1

Among the social network and information access factors, household head knowledge (awareness) of cluster farming is positively and significantly related to cluster participation. This could imply that raising awareness is an important activity for informing households about the benefits of participating in the cluster initiative. The other variable is neighbour participation in cluster farming. Surprisingly, neighbour participation is negatively related to cluster participation. This is contrary to our expectation as neighbour participation in cluster may avail more information to household at a lower cost and smaller distance. In a related study, Joffre et al. [8] indicated that interaction with neighbours may not have such a significant effect on household participation in group initiative, as it may even have negative effects in some cases due to possible free riding behaviours. On other hand, both awareness and neighbour variables become insignificant conditional on participation decision. This suggests that while such information networks are important in the initial stage when farmers want to know more about cluster farming and its benefits, once they join the cluster, they may need less of it, or membership in cluster itself provides them with sufficient information to decide whether or not to contribute more land.

Other important source of information for farm household is access to extension services. Household access to extension services is positively and significantly related to cluster participation, which may imply that extension services have played a significant role in the promotion of cluster farming initiative among rural farm households. Similar results were obtained in other related studies by Joffre et al. [8] and Tabe-Ojong et al. [38]. The result may suggest the need to use extension programs to raise awareness and encourage smallholder farmers to participate. However, this result also turns insignificant conditional on participation.

The other two interesting variables which are only used in second stage equations are cluster land size and number of cluster member farmers. Cluster land size is positively and significantly associated with the amount of land contributed conditional on participation decision. On other hand, the number of farmers per cluster farming is negatively related to the household contribution of land conditional on participation decision. Cluster land area variable may imply large clusters in terms of land size can achieve higher economies of scale with lower transaction costs and higher internal cohesion than large clusters with a large number of members. For example, it may be easier for the government to provide services such as mechanisation (e.g., ploughing tractors) to larger cluster areas with fewer members. This means that clusters with a larger area are more likely to attract new farmers to join or existing farmers to contribute more land. Related studies by Fischer and Qaim [54] and Markelova et al. [55] also indicated that while a larger group can achieve better economies of scale, a smaller group is easier to coordinate and monitor. In general, the results suggest that the government should pay attention to both cluster area and number of participants when forming new clusters or expanding existing clusters.

### 5.4 Robustness of result

We perform a couple of additional analysis and robustness checks to further confirm and corroborate our findings. Given the limitations of the nonlinear estimation especially when it comes to identification by functional form [56], we follow Tabe-Ojong et al. [57] in estimating linear models to relax this identification limitation. We use limited linear probability model to estimate our first stage probit model. The results as shown in S1 Table are similar in magnitudes, signs and statistical significance with the baseline nonlinear models. This confirms that the empirical findings and identification based on the first stage nonlinear probit model is not sensitive to the choice of functional form. The second robustness check was performed by estimating Tobit model using share of land contributed as different cluster farming measurements to confirm the results obtained from the DH model. In this model, we use the share of land contributed to cluster farming by households as our dependent variable. We again obtain similar results in this case, further supporting the results of relationship between farm size and cluster farming (see S2 Table in Supplementary information).

## 6. Conclusion and policy implications

The study uses cross-sectional data from 3,969 farm households in four main regions of Ethiopia to examine the relationship between farm size and cluster farming. We employ a two-stage double hurdle framework to assess this relationship in terms of farm households’ participation decisions in cluster farming and the extent to which they participate. By extent of participation, we mean the amount of land farm households contribute to cultivate under cluster farming. Our findings suggest that cluster farming is increasing with landholding size up to a certain threshold beyond which it declines, implying that land may be a binding constraint to the participation of farmers in the clusters. However, we find negative relationship for land size square term. This may imply that the positive relationship between land ownership and cluster farming may not hold for large farms. We also explore other factors such as farm household human capital related characteristics, access to information and social networks, access to institutional services and cluster-level characteristics. Our finding indicates that both individual household-level and cluster-level characteristics may play potential role in promoting cluster farming approach. We use different robustness estimation techniques to confirm the relationship between land ownership and cluster farming. Our findings provide evidence of crucial role of ensuring smallholder farmers access to farmland in order to promote entry into cluster farming and scale up the approach.

Given the findings from the study, our policy implications revolve around the importance of ensuring smallholder farmers’ land access in order to encourage their participation in cluster farming. However, land issues in Ethiopia are highly contestable and current land policies impose a number of restrictions on smallholders’ access to land and general land availability. For example, farmland is mainly acquired through inheritance, and farm households have access to only small plots of land. However, once the farm household children establish their own family units, families divide land plots among themselves, continuously dwindling land size. Furthermore, several land lease and large-scale investment developments also pose threat to smallholder land security and overall fertile land availability in the country. In many rural areas, large tracts of land are leased to privates, while increasing urbanization evicts farm households from their land, leaving them without equally available fertile land for farming.

Under this circumstance, granting smallholders access to farmland may boost cluster farming in Ethiopia. Literature shows land ownership and security can be ensured through land certification, registered title, or other means that reduce perceived insecurity of land tenure and possibly encourage farmer’s incentives to invest in land improving technologies and management systems. This may also be an essential mechanism to increase land use efficiency and improve overall smallholder livelihoods. Another policy consideration is improving Ethiopia’s land rental markets. Land markets such as land rent or crop-sharing could facilitate land transfer from relatively old, resource-poor farmers to younger, healthier, and more resource-rich farmers, thereby improving land allocative efficiency. These efforts may increase farm households’ and youths’ (who tend to more participate in cluster) access to land, facilitating their participation in cluster farming. Furthermore, policies regarding land leases and large-scale investments should be reconsidered from the perspective of smallholders, directly and meaningfully involving the local community. Cluster farming, on the other hand, has the potential to be used as a policy tool to assist smallholders in making better use of available land. The approach not only promotes economies of scale and facilitates targeted support from public and private actors, but it also encourages land consolidation by reducing widespread land fragmentation, as well as best farming practices that promote soil fertility and recommended input applications, among other things. Therefore, cluster farming itself could be a policy to facilitate farmers to transition from fragmented small-scale to larger-scale commercial farming.

The study has its own limitations. First, the study used cross-sectional data making causal inferences difficult although landholding size is assumed exogenous in this study. Second, as context is always important, caution should be made when drawing generalizations from the analysis. Nonetheless, the study’s findings could be generalized to developing countries considering that the agricultural landscape in Ethiopia closely mirrors various other nations where smallholder agriculture forms the backbone of the economy. That said, this is one of the first attempts to understand the relationship between land ownership and cluster farming. Future studies conducted on this premise are encouraged to enhance the external validity of the findings.

## Supporting information

S1 Table Estimates of linear probability model. (DOCX)

S2 Table Estimates of DHs with share of land contributed as dependent variable. (DOCX)

S1 Data (DTA)
