# Supplementary material for: Land as a binding constraint to cluster-based development in Ethiopia: To cluster or not to cluster?
Source: PLoS One. 2024 Apr 16;19(4):e0298784. doi: 10.1371/journal.pone.0298784 (PMC11020760; doi:10.1371/journal.pone.0298784)
Supplement: S1 Table — (DOCX) [file pone.0298784.s001.docx]

**Table S1. Estimates of Linear Probability Model**

|  | **Probit model Estimator (Probit)** | **Linear Probability Model (LPM)** |
| --- | --- | --- |
|  |  |  |
| HH total Landholding (Ha) | 0.20*** | 0.03*** |
|  | (0.03) | (0.01) |
| HH land holding square (Ha sq) | -0.01* | -0.00** |
|  | (0.00) | (0.00) |
| HH head's Age (Years) | -0.01*** | -0.00*** |
|  | (0.00) | (0.00) |
| HH primary education (Yes =1) | 0.35*** | 0.05*** |
|  | (0.06) | (0.01) |
| HH is female (Yes =1) | 0.61*** | 0.12*** |
|  | (0.09) | (0.02) |
| Neighbourhood participation (Yes =1) | -0.02 | -0.02 |
|  | (0.06) | (0.01) |
| Cluster existence awareness (Yes =1) | 1.70*** | 0.28*** |
|  | (0.08) | (0.01) |
| Group membership (Yes =1) | -0.01 | 0.02 |
|  | (0.06) | (0.01) |
| HH family size | 0.04*** | 0.01** |
|  | (0.01) | (0.00) |
| Access to storage facilities (Yes =1) | 0.31*** | 0.06*** |
|  | (0.06) | (0.01) |
| Access to credit services (Yes =1) | 1.25*** | 0.22*** |
|  | (0.06) | (0.01) |
| Access to extension services (Yes =1) | 0.88*** | 0.11*** |
|  | (0.12) | (0.02) |
| Off-farm activities (Yes =1) | 0.06 | 0.01 |
|  | (0.06) | (0.01) |
| Cluster total area size (ha) |  | 0.01*** |
|  |  | (0.00) |
| Cluster member size (number) |  | 0.01*** (0.00) |
| Crop dummies | Yes | Yes |
| Region dummies | Yes | Yes |
| Time controls | Yes | Yes |
| Constant | Yes | Yes |
|  |  |  |
| Observations | 3,559 | 3,559 |

Standard errors in parentheses *** p<0.01, ** p<0.05, * p<0.1
