# Supplementary material for: Land as a binding constraint to cluster-based development in Ethiopia: To cluster or not to cluster?
Source: PLoS One. 2024 Apr 16;19(4):e0298784. doi: 10.1371/journal.pone.0298784 (PMC11020760; doi:10.1371/journal.pone.0298784)
Supplement: S2 Table — (DOCX) [file pone.0298784.s002.docx]

**Table S2. Estimates of DHs with share of land contributed as dependent variable**

|  | **DH Model** | | **Tobit Model** |
| --- | --- | --- | --- |
|  | **I** | **II** | **III** |
|  |  |  |  |
| HH total Landholding (Ha) | 0.047*** | -0.095*** | -0.03*** |
|  | (0.008) | (0.006) | (0.01) |
| HH land holding square (Ha sq) | -0.001* | 0.004*** | 0.00 |
|  | (0.001) | (0.000) | (0.00) |
| HH head's Age (Years) | -0.003*** | -0.002*** | -0.00*** |
|  | (0.001) | (0.001) | (0.00) |
| HH primary education (Yes =1) | 0.084*** | 0.008 | 0.06*** |
|  | (0.015) | (0.015) | (0.02) |
| HH is female (Yes =1) | 0.147*** | 0.015 | 0.17*** |
|  | (0.022) | (0.018) | (0.02) |
| Neighbourhood participation (Yes =1) | -0.005 | 0.002 | -0.01 |
|  | (0.014) | (0.003) | (0.02) |
| Cluster existence awareness (Yes =1) | 0.406*** | -0.002** | 0.53*** |
|  | (0.017) | (0.012) | (0.03) |
| Group membership (Yes =1) | 0.147*** | 0.112** | -0.01 |
|  | (0.022) | (0.034) | (0.02) |
| HH family size | -0.002 | -0.007 | 0.01*** |
|  | (0.013) | (0.014) | (0.00) |
| Access to storage facilities (Yes =1) | 0.011*** | -0.056*** | 0.03** |
|  | (0.003) | (0.014) | (0.02) |
| Access to credit services (Yes =1) | 0.074*** | -0.0224 | 0.19*** |
|  | (0.014) | (0.012) | (0.02) |
| Access to extension services (Yes =1) | 0.300*** | -0.029 | 0.26*** |
|  | (0.012) | (0.039) | (0.04) |
| Off-farm activities (Yes =1) | 0.210*** | -0.037 | -0.01 |
|  | (0.028) | (0.012) | (0.02) |
| Cluster total area size (ha) |  | 0.004*** | 0.01*** |
|  |  | (0.000) | (0.00) |
| Cluster member size (number) |  | -0.002*** | 0.00*** |
|  |  | (0.000) | (0.00) |
| Crop dummies | Yes | Yes | Yes |
| Region dummies | Yes | Yes | Yes |
| Time controls | Yes | Yes | Yes |
| Constant | Yes | Yes | Yes |
|  |  |  |  |
| Observations | 3,559 | 1,853 | 3,559 |

Standard errors in parentheses *** p<0.01, ** p< 0.05, * p<0.1
